# Taming Membranes: Functional Immobilization of Biological Membranes in Hydrogels

**DOI:** 10.1371/journal.pone.0020435

**Published:** 2011-05-31

**Authors:** Ilja Kusters, Nobina Mukherjee, Menno R. de Jong, Sander Tans, Armağan Koçer, Arnold J. M. Driessen

**Affiliations:** 1 Department of Molecular Microbiology, Groningen Biomolecular Sciences and Biotechnology Institute and the Zernike Institute for Advanced Materials, University of Groningen, AG Groningen, the Netherlands; 2 Department of Membrane Enzymology, Groningen Biomolecular Sciences and Biotechnology Institute, University of Groningen, Groningen, the Netherlands; 3 BiOMaDe Technology Foundation, Groningen, the Netherlands; 4 AMOLF Institute, Amsterdam, the Netherlands; Université de Technologie de Compiègne, France

## Abstract

Single molecule studies on membrane proteins embedded in their native environment are hampered by the intrinsic difficulty of immobilizing elastic and sensitive biological membranes without interfering with protein activity. Here, we present hydrogels composed of nano-scaled fibers as a generally applicable tool to immobilize biological membrane vesicles of various size and lipid composition. Importantly, membrane proteins immobilized in the hydrogel as well as soluble proteins are fully active. The triggered opening of the mechanosensitive channel of large conductance (MscL) reconstituted in giant unilamellar vesicles (GUVs) was followed in time on single GUVs. Thus, kinetic studies of vectorial transport processes across biological membranes can be assessed on single, hydrogel immobilized, GUVs. Furthermore, protein translocation activity by the membrane embedded protein conducting channel of bacteria, SecYEG, in association with the soluble motor protein SecA was quantitatively assessed in bulk and at the single vesicle level in the hydrogel. This technique provides a new way to investigate membrane proteins in their native environment at the single molecule level by means of fluorescence microscopy.

## Introduction

Biological membranes are ubiquitous in all living cells and harbor a unique set of proteins, the membrane proteins. These play a crucial role in critical cellular and physiological processes such as nutrient transport, signaling, and energy-transduction. About 60% of the human druggable targets are membrane proteins [1]. Despite their importance, the knowledge of the function of membrane proteins lags far behind that of water-soluble proteins. This is due to the fragility of the lipid membrane rendering investigations of membrane proteins in their native environment challenging. At the same time, the high hydrophobicity and tendency to precipitate when extracted from the membrane environment make membrane proteins intrinsically difficult to analyze and handle. A true understanding of membrane protein functioning requires both ensemble and single molecule techniques such as fluorescence spectroscopy [2]. Single-molecule fluorescent spectroscopy methods are non-invasive tools that can be used to investigate protein functioning without the averaging of temporal or population heterogeneity from bulk experiments [3]. Hence, detailed kinetic information, intermediates and rate limiting steps of protein performance can be acquired [4]. For time resolved fluorescence spectroscopy, however, the membrane proteins need to remain in the observation area of a microscope, thus requiring immobilization of membrane protein or entire membranes. Immobilization of biological membranes without affecting the functionality of embedded membrane proteins has been a major challenge up to date due to the fragility of the lipid bilayer. Therefore, new immobilization techniques for the detailed investigation of membrane protein functioning at the single molecule level are crucial.

Surface supported planar lipid-bilayers have been developed to mimic cell membranes and accommodate membrane proteins. They allow the lateral mobility of lipids by a thin layer of water (1–2 nm) between the surface and the lipid bilayer [5]. However, this layer is insufficient to accommodate large soluble loops of membrane proteins and contact with the surface might lead to immobilization and/or even inactivation of the protein. To minimize the interaction with the supporting material, polymers [6] and hydrogels [7] were developed to cushion the bilayer. These polymer supported lipid bilayers improve the lateral mobility of reconstituted membrane proteins [6], [8]. Unfortunately, the formation of supported lipid bilayers is limited to certain lipid compositions that promote vesicle fusion and rupture on the supporting material [9]. However, lipids determine the physical and chemical properties of the membrane and may affect membrane proteins in their mobility and function. Lipids were also shown to directly control membrane proteins through specific interactions [10]. Moreover, for this method the required surface modifications and cleaning procedures are time consuming and costly. Thus, more versatile methods are required to study membrane proteins in immobilized membranes.

Surface immobilization of small (50–200 nm) unilamellar vesicles would allow to investigate the functioning of even a single membrane protein over a desired period of time. As these small membrane vesicles appear as diffraction limited spots in a fluorescence microscope, the membrane protein(s) are retained in the observation area albeit diffusing laterally in the lipid bilayer of the vesicles. Possibly due to incompatibility of membranes and embedded membrane proteins with the surfaces, which may result from multiple interactions, these studies are extremely rare. Recently, proteoliposomes (PLs) containing SecYEG channels were immobilized on a surface supported lipid bilayer [11]. However, the activity of the membrane channel was not definitely proven and the method was poorly documented.

Another approach to do single molecule studies on membrane proteins is by generating giant unilamellar vesicles (GUVs) with embedded membrane protein of interest and keeping them in the observation area for a desired period of time. (for review see [12]). Due to their big size (1–15 µm) GUVs have a low curvature and their membrane surface appears practically planar in the observation area of a confocal microscope. Thus, they offer a valuable tool to study the diffusion and oligomeric state of membrane proteins by fluorescence cross-correlation spectroscopy (FCCS). In addition, transport processes across the membranes of these giant vesicles can be investigated by high-resolution fluorescence imaging. However the same property, the size, makes the surface immobilization of GUVs very challenging as they have more available area that can interact with the surface. In most cases direct attachment of GUVs into a surface via surface modifications results in rupture and collapse of the GUV [13]. Besides, their size makes them more fragile to mechanical disturbances in the environment.

An ideal environment for the study of membrane proteins should therefore support a variety of biological membranes without inhibiting the functioning of the membrane-embedded proteins and at the same time should allow single molecule fluorescence measurements by keeping them in the observation area for a desired period of time. The immobilizing material should minimize interactions with the membrane surface and allow the embedded proteins to diffuse freely. As many membrane proteins interact with ligands or substrates, molecules of a wide sizes range should diffuse freely and access the entire membrane surface. At the same time, membrane vesicles of various sizes ranging from small (50–200 nm) up to several micron sized GUVs should be stably immobilized while maintaining their membrane integrity.

**Figure 1:**
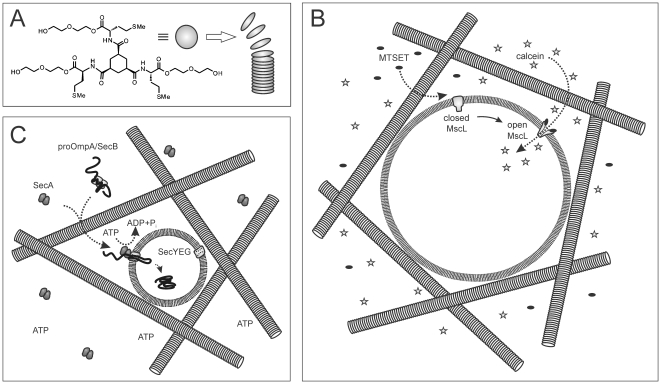
Hydrogels composed of self-assembling organic gelators immobilize membrane vesicles. (A) Gelator molecule 1 is based on 1,3,5-cyclohexyltricarboxamide and self-assembles into fibers of 20–100 nm. (B) Schematic representation of a hydrogel immobilized GUV with embedded MscL channel. The lipid bilayer is impermeable for the soluble fluorophore calcein when MscL occupies the closed conformation. Addition of MTSET triggers the opening of genetically engineered MscL and allows influx of calcein into the lumen of the GUV. (C) *In vitro* protein translocation into hydrogel immobilized PLs. SecA translocates the fluorescently labeled preprotein proOmpA through the SecYEG channel by multiple cycles of ATP hydrolysis. Inside the PL, proOmpA is protected against an externally added protease. Molecules in (B) and (C) are not drawn in scale.

In this study we present, for the first time, hydrogels composed of organic gelators for the functional immobilization of membrane proteins both in their native lipid environment and in synthetic lipid environments (PLs, GUVs). Hydrogels composed of self-assembling units of low-molecular-weight gelators based on 1,3,5-cyclohexyltricarboxamide form networks of nano-scaled fibers that are an attractive way to immobilize membrane vesicles [14]. The di-ethylene glycol functionalization of the gelator creates a low interacting fiber surface that minimizes surface interactions with biological molecules (Figure 1). Moreover, as fiber-fiber interactions are weak, the local mesh size of the gel is adjusted by the vesicles, allowing them to form their own cavity. Importantly, the vesicles are completely surrounded by an aqueous environment and the self-adapting mesh size of the gel allows the free diffusion of macromolecules such as proteins, yet restricts the movement of the membrane vesicle. The fibers of hydrogelators from the same family of gelators were previously shown to immobilize bilayer liposomes composed of zwitterionic synthetic lipids without interacting at the molecular level [14]. Here, we demonstrate that hydrogels based on 1,3,5-cyclohexyltricarboxamide effectively immobilize inverted membrane vesicles (IMVs) from *E. coli*, proteoliposomes (PLs) from native *E. coli* lipids and even several micron sized GUVs composed of a synthetic lipid mixture. The integrity of the lipid bilayer of the different vesicles remains intact during the immobilization procedure. Importantly, the embedded membrane proteins MscL, the mechanosensitive channel of large conductance of *E. coli*, and SecYEG, the protein-conducting channel of bacteria, are fully active. Since these hydrogels are optically transparent, they allow for fluorescent investigations of the activity of the membrane proteins and processes at the membrane interface down to the single molecule level.

## Results

### The membrane integrity of hydrogel immobilized (proteo-) liposomes is maintained

**Figure 2:**
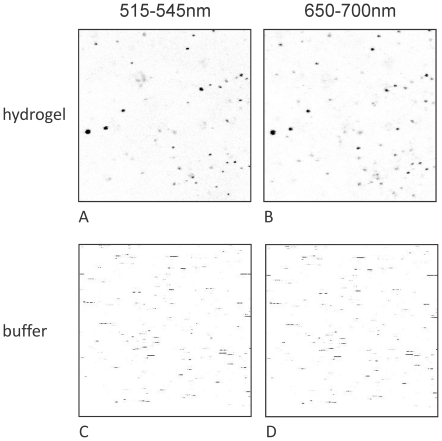
Liposomes containing fluorescent lipid analog DiD (maximum emission at 670 nm) filled with Alexa Fluor 488 (maximum emission at 517 nm) labeled glutathione, (A,B) hydrogel immobilized, (C,D) liposomes in suspension prior immobilization. Images were taken in a Dual-color laser-scanning confocal microscope by scanning an area of 30×30 µM in gel or solution.

Hydrogelator molecules based on 1,3,5-cyclohexyltricarboxamide self-assemble into nano-scaled fibers that form a three dimensional interpenetrating network with defined mesh size [14]. Here, we obtained hydrogels (Figure 1A) by cooling a hot solution (130°C) of 1.3% gelator 1 in buffer to room temperature. For visualization, liposomes of *E. coli* lipids supplemented with the fluorescent lipid analog DiD were filled with the water-soluble fluorophore Alexa Fluor 488 (AF488) attached to the tripeptide glutathione. Liposomes and proteoliposomes as prepared in this study typically have an average size of around 100 nm as observed by dynamic light scattering and NanoSight particle tracking (data not shown). Immobilization of the filled liposomes was achieved by short vortexing (vigorous shaking) of the liposome suspension in a 1∶1 ratio with the preformed gel. Subsequently, the gel was applied onto a microscopic cover slip followed by a resting phase in which the gel “heals”, i.e. crosslinks between fibers are re-formed leading to a network with incorporated liposomes. The immobilized liposomes were imaged in the gel using a dual color laser scanning confocal microscope. In contrast to the PLs in suspension (Figure 2C and D) which moved while taking the image, the location of the PLs in the hydrogel was entirely stable within the confocal plane (Figure 2A and B). Moreover, the fluorescent signals of the two fluorophores DiD and AF488 co-localize and the level of liposome encapsulated AF488 was similar to that of liposomes prior immobilization (Figure S1). The latter was determined by dual-color fluorescent-burst analysis (DCFBA, for review see [15]), a technique that enables the quantification of co-localizing fluorescent signals and the calculation of relative fluorescence ratios. Using DCFBA, the fluorescent intensities of encapsulated AF488 and DiD of individual liposomes were determined and the ratio of AF488/DiD was calculated for both liposomes diffusing in solution and immobilized in the hydrogel (Figure S1). The average ratio AF488/DiD is an arbitrary unit for the amount of encapsulated dye per liposome and was found to be similar for both conditions (Figure S1). Thus, no significant leakage of AF488 occurred during immobilization in the hydrogel.

### Opening of the mechanosensitive channel of large conductance (MscL) observed on a single GUV

**Figure 3:**
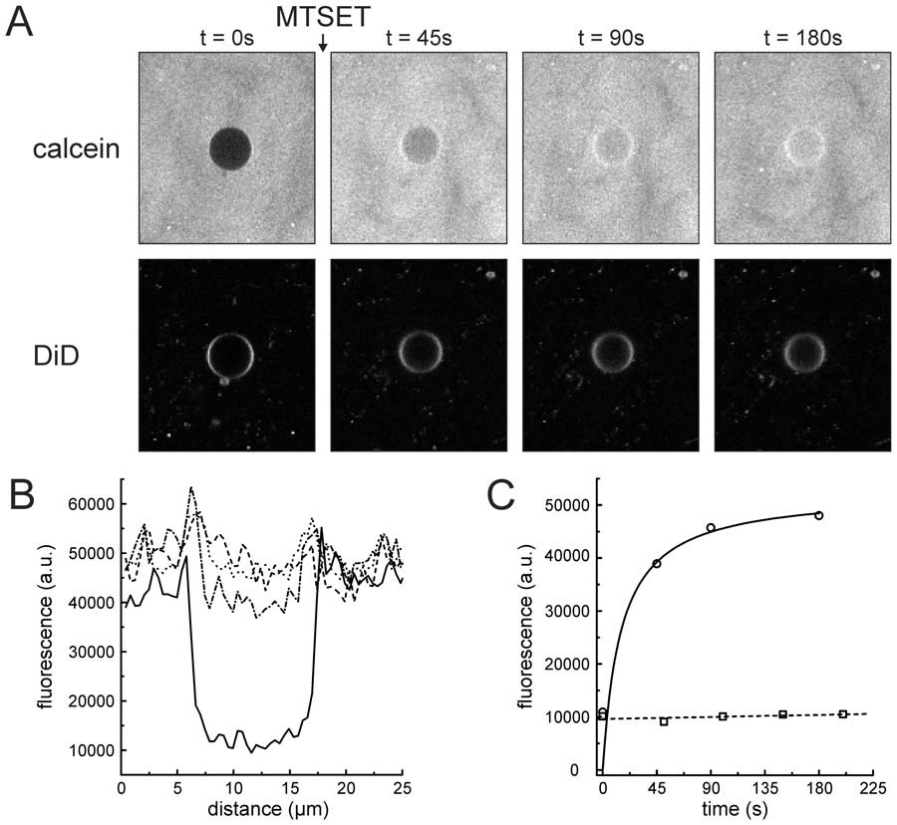
Opening of the MscL channel monitored on a single hydrogel immobilized GUV supplemented with the fluorescent lipid analog DiD. (A) Addition of MTSET to the top of the hydrogel causes opening of the membrane embedded MscL channel whereupon the soluble fluorophore calcein diffuses into the lumen of the GUV. (B) Calcein fluorescence inside and outside of the GUV quantified by cross-sections through the center of the GUV depicted in (A) at the different time points; t = 0 s (straight line), 45 s (dash-dot), 90 s (dot), 180 s (dash). (C) Calcein fluorescence at the center of the GUV (A) at the indicated time points (straight line). Background fluorescence inside a GUV (as shown in Fig. 5) did not change upon addition of buffer (dashed line).

To investigate the suitability of the hydrogel for immobilization of very large and fragile membrane systems, we prepared several micron sized GUVs and tested them for membrane integrity and membrane protein activity in the gel. The performance of a hydrogel as described in the previous section was tested for GUV immobilization and monitoring of membrane protein activity. The mechanosensitive channel of large conductance, MscL, from *E. coli* was reconstituted into liposomes and the resulting PLs were used to generate DiD labeled GUVs [16]. MscL is a bacterial membrane channel protein that senses the increase in the lateral pressure in the membrane due to a hypo-osmotic shock. It functions as a safety valve to release the turgor pressure by opening a large, non-selective pore and releasing molecules and even peptides up to 6.5 kDa [17], [18]. Here we used an engineered version of this channel, MscLG22C [19], [20]. MscLG22C can be activated by labeling the cysteine at position22 with the charged molecule [2-(trimethylammonium)ethyl]methane thiosulfonate bromide (MTSET). This covalent modification results in the opening and closing of the channel in the absence of its native trigger, i.e. membrane tension [21]. The performance of MscL, embedded in hydrogel immobilized GUVs, was tested by following the triggered influx of a fluorescent dye (calcein) into GUVs through MscL using confocal microscopy. For incorporation of GUVs into the gel, we took advantage of the inherent reversibility of the physical crosslinks that hold the gel together. By vigorous shaking on a vortex mixer, crosslinks between and within fibers are broken, resulting in a viscous suspension. Due to their fragile nature, DiD labeled GUVs containing MscL were embedded in a hydrogel of gelator 1 by first liquefying the preformed hydrogel by vigorous shaking it for 1 min and mixing the hydrogel afterwards with 0.5 volume of GUVs using a pipette. The gel-GUV mixture was applied on a cover slip and incubated for about 10 minutes to ensure gel healing. During this time period, new crosslinks between the fibers are formed and short fibers can assemble and form longer fibers. The DiD stained membrane of an approximately 10 µm sized MscL-GUV was imaged by a two-dimensional scan using a dual laser confocal microscope (Figure 3A, lower panel). Calcein, added to the hydrogel prior to imaging without disturbing the setup, is surrounding the GUVs (Figure 3A, upper panel, first picture). Since MscL was not activated at this point, it stayed closed and calcein could not penetrate into the GUV demonstrating the membrane integrity. Upon the addition of the membrane-impermeable channel activator, MTSET, the channel opened and the gradual time dependent filling up of the GUV was observed (Figure 3A–C). The concentration of calcein inside and outside of the GUV was quantified by a one dimensional cross-section through the center of the GUV at the different time points illustrating MscL channel opening (Figure 3B). Time dependent influx of calcein into the GUV was quantified measuring the calcein fluorescence at the center of the GUV resulting in a kinetic saturating curve (Figure 3C). Equilibration of the calcein concentrations inside and outside of the GUV was reached at around 180 s (Figure 3C), in accordance with the results obtained with PLs in solution (data not shown). Upon addition of buffer containing MTSET, a slight shift in the focal plane was observed resulting from a minor movement of the gel such that a smaller cross-section of the GUV was imaged. As a control, buffer without MTSET was added to MscL GUVs in another gel (Figure S2). No influx of calcein into the GUV could be observed (Figure 3C and S2B) demonstrating the stability of the GUV and the closed state of the MscL channel. Taken together, this data demonstrates that MscL, a mechanosensitive membrane protein, in GUVs is not affected by the hydrogel, which allows single molecule measurements. Moreover, different ways of vesicle immobilization are possible and ensure membrane integrity even of fragile, micron sized vesicles.

### The hydrogel does not affect protein-protein interactions

**Figure 4:**
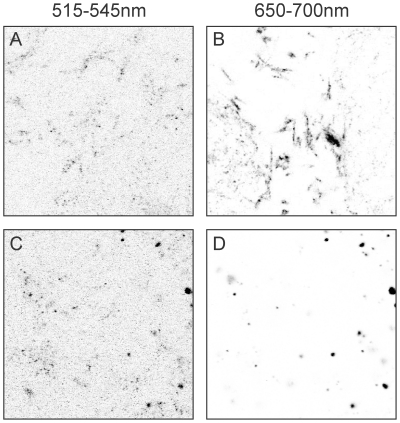
Protein interactions in the hydrogel. A fraction of SecA-FM (A) and proOmpA-Atto647N in complex with the chaperone SecB (B) bind non-specifically to the OG8 fibers. SecA-FM (C) binding to PLs containing SecYEG-Atto647N (D) is visible as co-localizing signal. In presence of SecYEG-PLs non-specific binding of SecA-FM to the gel fibers is eliminated (A,C).

Next, the compatibility of the hydrogel with fluorescently labeled proteins was tested. Here, we focus on the protein translocation system of *E. coli*. This system consists of a multi-protein complex termed ’translocase’ that includes a protein conducting channel, SecYEG, embedded in the cytoplasmic membrane and a motor protein, SecA (for review see [22]). Secretory proteins (preproteins) are translocated through the translocase by SecA through multiple cycles of ATP hydrolysis (Figure 1C) [23], [24], [25]. Protein translocation of fluorescently labeled preproteins into SecYEG containing IMVs or PLs can be followed in bulk by protease protection [26] and by the formation of a translocation intermediate that can be monitored by FRET between a donor-fluorophore on the trapped preprotein and an acceptor fluorophore attached to the exit of the SecYEG pore (A. Kedrov et al., submitted). Here, using the procedure described above, we mixed proteins and PLs into hydrogels containing 0.65% gelator 1. While GFP was found freely diffusing in the gel as determined by fluorescence cross correlation spectroscopy (FCS, data not shown), the very hydrophobic and partially unfolded model preprotein proOmpA labeled with Atto647N bound non-specifically to the gel fibers (Figure 4B). Furthermore, a fraction of the otherwise freely diffusing fluorescein labeled SecA (SecA-FM) interacted with the fibers in absence of SecYEG containing PLs (Figure 4A). However, the non-specific binding of SecA-FM was virtually absent in the presence of immobilized proteoliposomes containing SecYEG-Atto647N that bound SecA-FM specifically (compare Figure 4C and D). This is likely due to the lower concentration of soluble SecA as a large fraction of SecA is bound to the SecYEG-PLs suggesting that the non-specific interaction is dependent on the SecA concentration. Altogether, this data demonstrates that interaction of a membrane receptor (SecYEG) with its ligand (SecA) can be studied on single liposome level using hydrogel immobilized PLs or IMVs.

### Hydrogel immobilized PLs and IMVs are active in protein translocation

**Figure 5:**
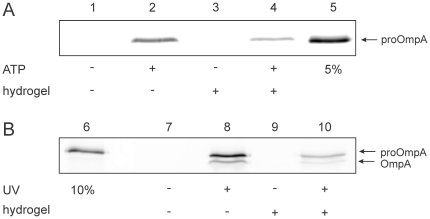
*In vitro* protein translocation in a hydrogel composed of gelator 1 and in suspension. Upon addition of ATP, SecA mediates the translocation of proOmpA into the PLs thereby rendering it resistant against an externally added protease (A). Translocation in the hydrogel or in suspension can be triggered by UV light when NPE-caged ATP is present (B). Lane 5, 6 serves as standard 5 and 10% of input material, respectively.

The activity of the translocase was examined by *in vitro* translocation of proOmpA-FM into SecYEG-PLs immobilized in a hydrogel composed of 0.65% gelator 1. ProOmpA-FM in complex with SecB, SecA, PLs and ATP were mixed simultaneously with the pre-formed hydrogel and incubated first at room temperature to heal the gel and then at 37°C. After 15 min, proteinase K was mixed with the gel to digest non-translocated proOmpA-FM. In order to collect translocated proOmpA, proteins in the gel were precipitated by addition of TCA and analyzed on SDS-PAGE using a fluorescence imager. Significant translocation activity in the hydrogel was observed after 15 min (Figure 5A, lane 4). Translocation activity was reduced to about 30% compared to translocation in suspension (Figure 5A, lane 2), possibly due to non-specific binding of the substrate proOmpA-Atto647N to the gel fibers (Figure 4B) preventing multiple turnovers of protein translocation. Since the healing of the gel and immobilization of the membranes is completed only after 1–2 min, *in vitro* protein translocation into fully immobilized *E. coli* IMVs was triggered by UV light induced un-caging of NPE-caged ATP (Figure 5B). Unlike the proteoliposomes, in IMVs, the membrane associated leader peptidase cleaves off the signal peptide resulting in the mature OmpA (Figure 5B). Similarly to the PLs, protein translocation activity of IMVs in the hydrogel was reduced to about 20% compared to translocation in solution after 15 min. However, for the first time, protein translocation was observed on immobilized vesicles in bulk. This data demonstrates that the translocon is functioning inside the gel, which is compatible with established biochemical methodologies such as TCA precipitation and SDS-gel electrophoresis.

### Protein translocation can be monitored at the single PL level by FRET

**Figure 6:**
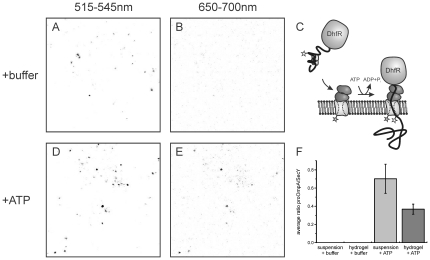
Activity of hydrogel immobilized PL monitored by DCFBA-FRET. Immobilized proteoliposomes containing SecYEG-FM were excited with a 488 nm laser only and fluorescence at 515–545 nm (A, D) and 650–700 nm (B, E) was recorded simultaneously. (A) PLs containing SecYEG-FM after addition of buffer. (B) The same area as (A) at 650–700 nm shows only background fluorescence. (C) Schematic representation of proOmpA-DhfR intermediate formation. In presence of ATP, SecA translocates the proOmpA domain until the tightly folded DhfR domain stalls translocation resulting in a stable complex. Atto647N on proOmpA serves as acceptor for FRET from a donor fluorophore attached to the exit of the SeYEG channel. (D) PLs containing SecYEG-FM after addition of ATP leading to formation of protein translocation intermediates of proOmpA-DhfR-Atto647N. (E) Resulting FRET signals appear as co-localizing fluorescence at 650–700 nm. (F) Quantification of FRET efficiencies by DCFBA in the hydrogel and in suspension.

Since the chaperone SecB keeps the model substrate proOmpA in a partially unfolded and therefore translocation competent state, substantial non specific interactions of proOmpA with membranes and other surfaces occur. This renders monitoring of protein translocation by means of proOmpA accumulation inside of small vesicles intrinsically difficult. Therefore, to quantitatively assess protein translocation activity at the single liposome level, we performed DCFBA-FRET experiments employing a dual color laser scanning confocal microscope. As proOmpA was labeled with the acceptor fluorophore Atto647N (647 nm/669 nm, excitation/emission) non specifically bound proOmpA was not visible when exciting the donor fluorophore with the 488 nm laser line. The ratio of donor and acceptor fluorescence intensity in a FRET experiment can be used to determine FRET efficiencies and by immobilizing PLs in the hydrogel, FRET efficiencies for individual liposomes can be obtained. Proteoliposomes containing fluorescein labeled SecYEG, SecA and the fusion protein proOmpA(C282)-DhfR-Atto647N in complex with SecB were immobilized in a hydrogel of gelator 1 as described in the previous sections (Figure 6). The DhfR domain of the proOmpA-DhfR fusion protein is tightly folded in presence of its ligands NADPH and methotraxate and translocation results in a stable translocation intermediate with the proOmpA-DhfR trapped in the SecYEG channel (Figure 6C) [27], [28]. Using fluorescently labeled preprotein and SecY, formation of the translocation intermediate is expected to result in FRET between the donor fluorophore on SecYEG and the acceptor on proOmpA(C282)-DhfR (Kedrov et al, submitted, Figure 6C). In order to monitor single liposome FRET of translocation intermediates, we performed confocal scans in the gel to record fluorescence signals for blue (donor fluorophore on SecY, Figure 6A and D) and red fluorescence (acceptor fluorophore on proOmpA-DhfR, Figure 6B and E) simultaneously, using the 488 nm laser only for donor excitation. Co-localizing signals were identified and quantified with DCFBA as the ratio of proOmpA (C282)-DhfR-Atto647N over SecYEG-FM fluorescence. This ratio serves as an arbitrary unit for the FRET efficiency per liposome. In absence of ATP, no ratio of Atto647N/FM could be determined as fluorescence intensities at 650–700 nm did not exceed background levels (Figure 6B and F). However, when ATP was added to the reaction, fluorescent signals at 650–700 nm appeared originating from FRET between fluorophores on SecY and proOmpA-DhfR (compare Figure 6D and E, respectively). The efficiency of translocation intermediate formation in the hydrogel as determined by the average FRET efficiency per liposome was ∼50% compared to suspension (Figure 6F). This further demonstrates that the translocon is functional when reconstituted in PLs that are immobilized in the hydrogel.

## Discussion

To immobilize membrane vesicles with a size ranging from 100 nm to several micrometers such as proteoliposomes (PLs), IMVs and GUVs, a new method employing hydrogels composed of self-assembling low molecular weight gelators was investigated. Here, we demonstrate that the nano-scaled fibers of hydrogels based on 1,3,5-cyclohexyltricarboxamide are efficiently immobilizing (proteo-) liposomes composed of *E. coli* lipids and synthetic lipid mixtures without disturbing membrane integrity. This is evident as the water soluble tripeptide glutathione labeled with AF488 remains inside the liposomes after immobilization. Furthermore, calcein is unable to penetrate hydrogel immobilized GUVs containing a closed MscL channel. In previous studies, leakage of fluorescently labeled glutathione from liposomes was studied in investigations on the pore forming mechanism of the antimicrobial peptide melittin [29] and on the pore size of the mechanosensitive channel MscL [18]. Thus, if membrane integrity had been compromised during liposome or GUV immobilization, significant leakage would have been observed.

The hydrogels presented in this study are inert to factors such as pH, salts and temperature allowing immobilization of membrane vesicles at all physiologically relevant conditions. As the mesh size of the gel fibers is adjusted by the dimensions of the encapsulated particles, membrane vesicles of various sizes can be incorporated. Importantly, the biological activity of embedded membrane proteins in hydrogel immobilized membrane vesicles is preserved. Thus, the hydrogels are to a high degree biologically orthogonal. The fast and easy gel immobilization further allows high throughput sample preparation and single molecule measurements without time consuming surface preparations. Several strategies can be employed to incorporate molecules and vesicles in the gel. While small membrane vesicles and molecules can be vortexed with the gel, immobilization of fragile GUVs is accomplished by first liquefying the gel following by mild mixing with the GUV containing suspension. Furthermore, small molecules can be incorporated in the gel by addition to the top of the gel upon which they diffuse throughout the gel. This is evident by the here presented triggered opening of the MscL channel upon addition of the activator MTSET to the top of the formed gel containing immobilized GUVs. Influx of the soluble fluorophore calcein into single GUVs was followed in time by confocal microscopy providing a new tool to the GUV study tool box and investigation of MscL function in detail and at the single GUV level. This data demonstrates that GUVs are powerful in investigating membrane transport processes as their size allows measurements inside their lumen using confocal microscopy. Moreover, hydrogel immobilization of GUVs is possible and allows measurements without interference from surfaces as molecules can diffuse freely in the gel and surround the GUVs from all sides.

Immobilization of small membrane vesicles such as PLs and IMVs allows tackling a different set of questions on membrane protein functioning as possible in GUVs. In the latter, due to size, the embedded membrane proteins can move out of the observation plane of a confocal microscope by lateral diffusion in the lipid bilayer. As PLs and IMVs appear as diffraction limited spots, membrane proteins cannot escape from the observation area. Thus, by downscaling the number of membrane proteins per liposome, single membrane proteins can be immobilized in their native environment. We performed *in vitro* protein translocation experiments with hydrogel immobilized PLs and IMVs, both in bulk and on the single vesicle level, and observed significant protein translocation activity although with reduced efficiency. The latter is most likely due to non-specific binding of the partially unfolded model substrate proOmpA to the hydrogel fibers by which multiple translocation events per channel are prevented. In order to determine protein translocation activity inside the hydrogel and on the single liposome level, laser scanning DCFBA was employed. DCFBA provides a high throughput tool to measure a large number of liposomes thus enabling improved statistics. The FRET efficiency per hydrogel immobilized PL was reduced to 50% compared to liposomes in solution. Trapping of proOmpA-DhfR inside SecYEG blocks the channel for further translocation. Thus, multiple turnovers of protein translocation are prevented and the withdrawal of substrate due to non-specific binding to the fibers may be less severe. It appears, however, that proOmpA-DhfR bound to SecYEG prior immobilization may be ripped off from the PLs during mixing with the gel resulting in a reduction of intermediates. This data, however, demonstrates that hydrogels enable single molecule studies on membrane proteins incorporated in native or synthetic lipid mixtures.

In summary, the biological activity of (folded) proteins in the gel is sustained and macromolecules can diffuse freely in the gel due to its large mesh size. The transparency of the hydrogel allows the application of optical microscopy and processes at the membrane interface or within can be quantitatively assessed at the single vesicle and single molecule level. Thus, hydrogels composed of nano-scaled fibers are a new, generally applicable, immobilization tool for a wide range of native and synthetic membrane systems for the study of membrane proteins, their functioning and interaction with ligands from bulk down to the single molecule level.

## Material and Methods

### Hydrogels

Gelator 1 was synthesized as described in [14]. Preformed hydrogels were obtained by solubilizing 1.3% (w/v) gelator 1 in buffer (50 mM Hepes-KOH, 30 mM KCl, and 2 mM MgCl_2_) by heating to 130°C using an oil bath. After cooling to <100°C, the hot suspension was aliquoted to 50 µl and cooled down to room temperature (RT) upon which the gels formed. Incorporation and immobilization of IMVs or PLs was achieved by a short vortexing with one volume IMV/PL containing buffer.

### Preparation of ‘filled’ liposomes, SecYEG reconstitution and *in vitro* translocation

SecYEG was purified, fluorescently labeled and reconstituted as described [26]. Filling of liposomes was achieved by a procedure similar to SecYEG reconstitution. Addition of 10 µM AlexaFluor488-glutathione (AF488-glu) to detergent solubilized (0.5% Triton X-100/0.2% n-Dodecyl-β-maltoside) lipids supplemented with DiD (1∶100,000 molar ratio) and subsequent detergent removal by polystyrene beads (Bio-Beads SM-2 adsorbents) lead to the formation of AF488-gluthatione filled liposomes. Due to the absorption of AF488-glu to the polystyrene beads, only a fraction of the AF488-glu was encapsulated and virtually no free fluorophore was detected. IMVs, SecA, SecB, proOmpA, proOmpA-DhfR were purified as described elsewhere [26]. *In vitro* protein translocation was essentially performed as described previously [26]. Shortly, after addition or un-caging (2 min of UV, 360 nm, 4W lamp) of ATP the translocation reaction was incubated at 37°C for 15 min. Subsequently, proteinase K was added to digest not translocated proOmpA. After 15 min incubation at 37°C, proteins were precipitated by addition of 10% TCA (final concentration) for 30 min on ice. Prior centrifugation (16000× g, 15 min) ten volumes of a 4∶1 aceton-water mixture were added to the TCA precipitation to dissolve the organic gelator. The pellet was washed once with the acetone-water mixture, dried at 37°C and dissolved in SDS-sample buffer before it was applied to SDS-PAGE. The fluorescently labeled proOmpA was visualized in a gel imager (Fuji).

### Formation of GUVs containing MscL channels

Phosphatidylcholine (DPhPC, 1,2-Diphytanoyl-sn-Glycero-3-Phosphocholine) and POPG (PG, 1-Palmitoyl-2-Oleoyl-sn-Glycero-3-[Phospho-rac-(1-glycerol)], Sodium salt) were purchased from Avanti polar lipids. DPhPC was chosen as the PC component for our lipid composition for its high mechanical and chemical stability. Cholesterol (3β-Hydroxy-5-cholestene, C27H46O) was obtained from Sigma. MTSET ([2-(trimethylammonio)ethyl] methanethiosulfonate and bio-beads (Bio-Beads SM-2 adsorbents) were from Anatrace and Bio-Rad Laboratories B.V., respectively. Giant unilamellar vesicles (5-20 µm) were formed from PLs (150 nm) by electroformation as described previously [16]. Briefly, first MscL was produced, isolated and reconstituted into DPhPC:POPG:cholesterol liposomes (70∶25∶5 weight ratio) by a detergent-mediated reconstitution method as indicated before [19], [20]. The resulting PLs were used to obtain a thin lipid film on the surface of conductive glass slides (ITO). Droplets of 2 µl containing 0.8 mg/ml PLs in 2 mM MOPS-Tris buffer pH 7.0 were applied to the glass surface followed by overnight partial dehydration in a vacuum desiccator at 4°C. After the PL films were obtained, an electroformation chamber was prepared in NanionVecicle Prep Pro (Nanion Technologies GmbH, Munich, Germany). The chamber was filled with 250 mM sucrose. An AC voltage was applied for 4 hours across the cell unit with stepwise increases from 0.1 to 1.1 V at 12 kHz frequency. At the end, in order to detach glass attached giant unilamelar liposomes, the AC current was lowered to 4 Hz and voltage raised to 2 V for 30 min. Vesicles formed in this way had a diameter of 10–15 µm. In order to image the GUVs, a fluorescent lipid analogue, the fluorescent lipid analog DiD (650 nm/670 nm, excitation/emission) was added to the vesicles after they were formed. Calcein (497 nm/516 nm, excitation/emission) was used as an external dye for uptake by the GUVs.

### Fluorescent measurements

Immobilized (proteo-) liposomes were imaged in a dual-color laser scanning confocal microscope that is described in detail elsewhere [18], [29]. The two laser beams (488 nm, argon ion laser; 633 nm, He-Ne laser) were aligned to a high degree of spatial overlap and moved simultaneously with a galvanometer optical scanner through the sample. Two confocal images (30×30 µm) of the blue and red fluorescence channels were recorded simultaneously. For the laser-scanning dual-color fluorescent burst analysis (lsDCFBA, for review see [15]) measurements, fluorescent bursts from the spectrally well separated fluorophores (AF488/DiD or FM/Atto647N) were identified using arbitrary offsets as described [15]. The ratio of the fluorescence of one fluorophore over the other (AF488/DiD and Atto647N/FM, respectively) is an arbitrary unit for the relative stoichiometry of both fluorophore. The DCFBA-software can be downloaded at www.bogeert.com/DCFBA/publish.htm. Hydrogel immobilized GUVs and calcein were imaged in a Confocor3 confocal microscope (Zeiss) using the 488 nm argon ion laser and the 633 nm He-Ne laser.

## Supporting Information

Figure S1 **Membrane integrity of liposomes remains intact during hydrogel immobilization.** DCFBA analysis of liposomes filled with the soluble fluorophore AF488-glutathione and supplemented with the fluorescent lipid analog DiD determines the relative stoichiometry of the co-localizing fluorophores (for review see [2]). Hydrogel immobilized liposomes had comparable amounts of AF488 encapsulated as liposomes prior immobilization.
(TIF)

Figure S2 **The MscL channel remains in its closed state in absence of a trigger.** (A) MscL containing and DiD stained GUV at various time points after addition of buffer. (B) Calcein fluorescence outside and inside the GUV was quantified by cross-sections through the center of the GUV depicted in A at the different time points; t = 0 s (straight line), 50 s (dash-dot), 100 s (dot), 200 s (dash).
(TIF)
