# Supplementary figures and images for: Taming Membranes: Functional Immobilization of Biological Membranes in Hydrogels
Source: PLoS One. 2011 May 31;6(5):e20435. doi: 10.1371/journal.pone.0020435 (PMC3105061; doi:10.1371/journal.pone.0020435)

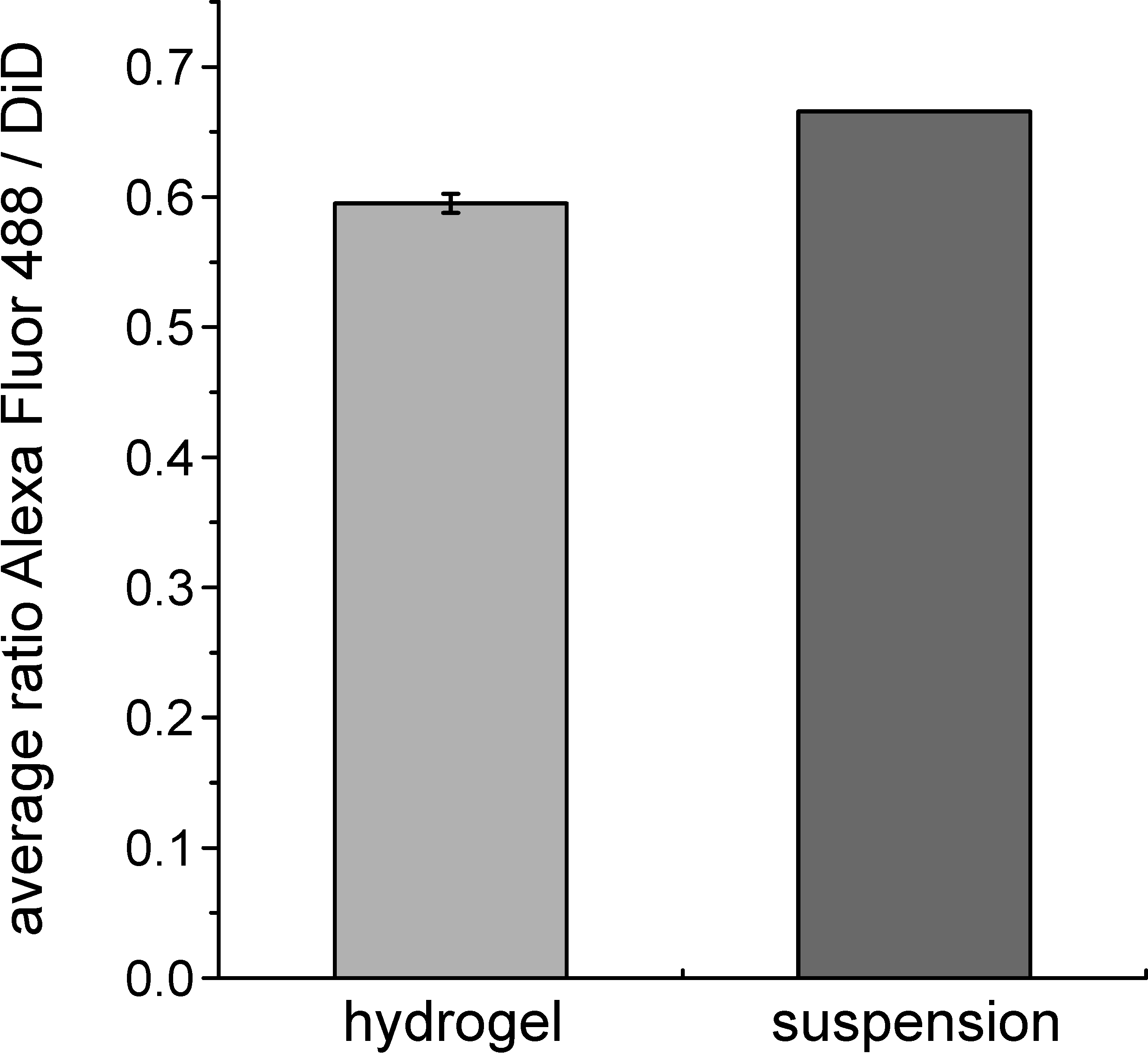

Supplement: Figure S1 — Membrane integrity of liposomes remains intact during hydrogel immobilization. DCFBA analysis of liposomes filled with the soluble fluorophore AF488-glutathione and supplemented with the fluorescent lipid analog DiD determines the relative stoichiometry of the co-localizing fluorophores (for review see [2]). Hydrogel immobilized liposomes had comparable amounts of AF488 encapsulated as liposomes prior immobilization. (TIF) [file pone.0020435.s001.tif]

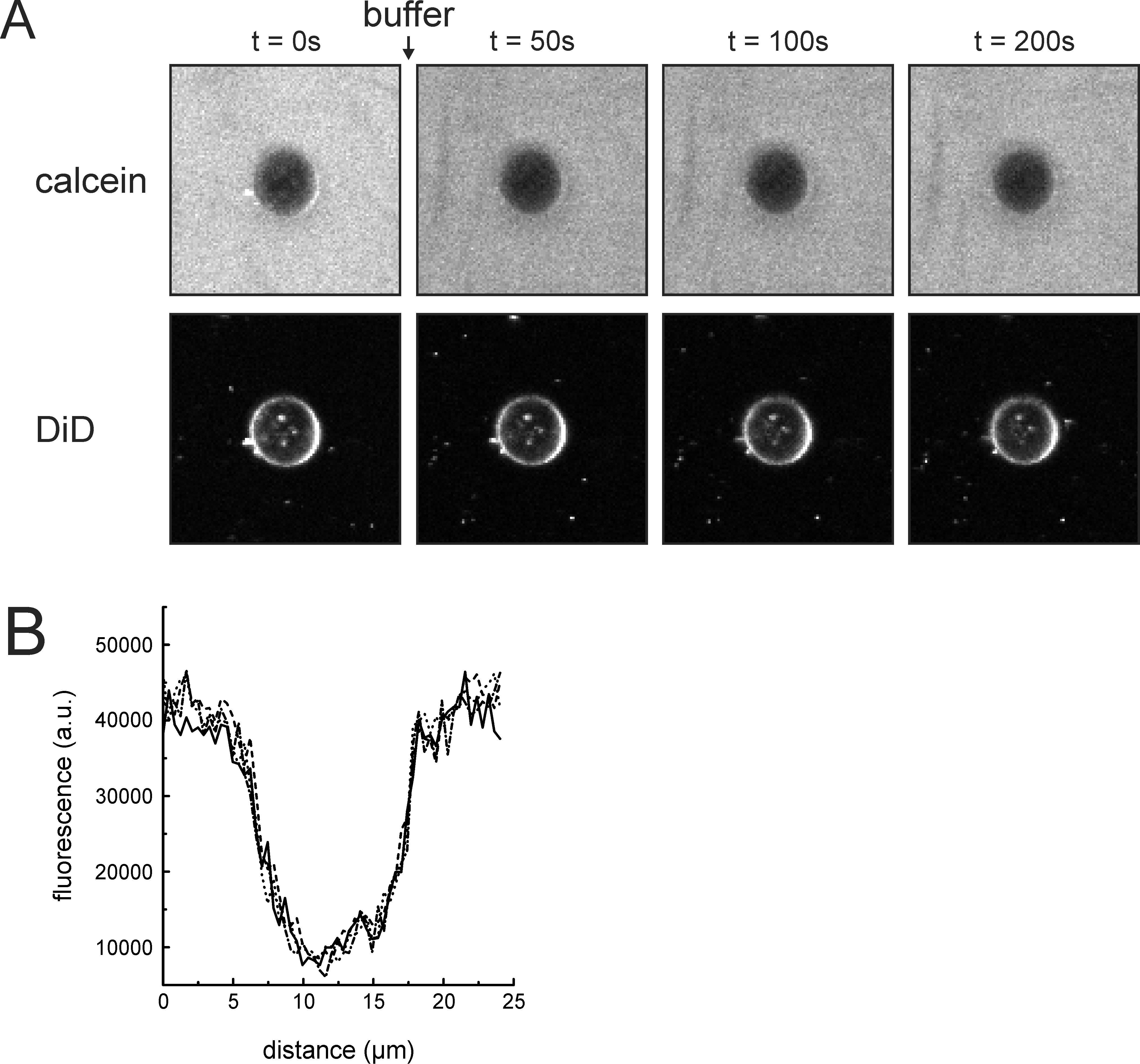

Supplement: Figure S2 — The MscL channel remains in its closed state in absence of a trigger. (A) MscL containing and DiD stained GUV at various time points after addition of buffer. (B) Calcein fluorescence outside and inside the GUV was quantified by cross-sections through the center of the GUV depicted in A at the different time points; t = 0 s (straight line), 50 s (dash-dot), 100 s (dot), 200 s (dash). (TIF) [file pone.0020435.s002.tif]
